# Design and Implementation of a Digitally Enabled Care Pathway to Improve Management of Depression in a Large Health Care System: Protocol for the Implementation of a Patient Care Platform

**DOI:** 10.2196/43788

**Published:** 2023-06-23

**Authors:** Rasha Khatib, Maggie McCue, Chris Blair, Anit Roy, John Franco, Ben Fehnert, James King, Sara Sarkey, Lambros Chrones, Michael Martin, Christopher Kabir, David E Kemp

**Affiliations:** 1 Advocate Aurora Research Institute Advocate Health Milwaukee, WI United States; 2 Takeda Pharmaceuticals U.S.A., Inc. Lexington, MA United States; 3 Advocate Aurora Health Downers Grove, IL United States; 4 Cognition Kit Cambridge United Kingdom; 5 Ctrl Group / Fora Health London United Kingdom; 6 Advocate Research Institute Advocate Health Care Downers Grove, IL United States

**Keywords:** mental health, depression, depressive, major depressive disorder, depression management, primary care, patient engagement, measurement-based care, shared decision-making, user-centered design, mobile app, digital platform, mobile phone, mobile health, adherence

## Abstract

**Background:**

Major depressive disorder (MDD) is a serious public health concern worldwide. A treatment approach that incorporates measurement-based care (MBC) and shared decision-making between patients with MDD and their providers may foster patient engagement and improve clinical outcomes. While digital tools such as mobile apps show promise for expanding health interventions, these apps are rarely integrated into clinical practice.

**Objective:**

The primary objective of this ongoing study is to determine whether implementation of a digital tool—the *Pathway Platform*—in primary care improves adherence to MBC practices; here, we present the study methods.

**Methods:**

This large-scale, real-world implementation study is based on a pilot study of an earlier iteration of a mobile app (the *Pathway app*) that confirmed the feasibility of using the app in patients with MDD and showed a positive trend in patient engagement in the app arm. In addition, a user-centered design approach that included qualitative assessments from patients and providers was used to improve understanding of the patient journey and care team workflows. User feedback highlighted the need for enhanced features, education modules, and real-time data sharing via integration with the electronic health record. The current iteration of the *Platform* includes the newest version of the *Pathway app*, education modules for both patients and providers, and real-time patient-level data sharing with the electronic health record. The study takes place in primary care sites within the Advocate Aurora Health system in Illinois and includes adult patients with MDD who were recently prescribed monotherapy antidepressant medication (defined as a new start, medication switch, or dose change in the past 3 months). Clinical performance and selected patient outcomes will be compared before and after the implementation of the *Platform*.

**Results:**

Patient recruitment was completed in July 2022, with initial results expected in mid-2023.

**Conclusions:**

This study will provide useful insights into real-world integration of a digital platform within a large health system. The methods presented here highlight the unique user-centric development of the *Pathway Platform*, which has resulted in an enhanced digital tool with the potential to foster MBC and shared decision-making, improve patient-provider communication, and ultimately lead to optimized treatment outcomes for patients with MDD.

**Trial Registration:**

ClinicalTrials.gov NCT04891224; https://clinicaltrials.gov/ct2/show/NCT04891224

**International Registered Report Identifier (IRRID):**

DERR1-10.2196/43788

## Introduction

### Background

Major depressive disorder (MDD) is a common recurring mental health disorder and a serious public health concern worldwide [1,2]. In 2019, an estimated 19.4 million adults in the United States had at least 1 major depressive episode, which represents 7.8% of the adult US population [3]. The economic burden of MDD for 2020 in the United States was estimated at US $326.2 billion, with approximately 73.2% of the cost attributed to impairment in role function [4,5]. Many factors complicate the treatment of MDD, including multiple antidepressant therapy options, the importance of adherence and adequate duration of therapy to optimize response, and the variation commonly seen in time to response and medication tolerability among patients. American Psychiatric Association MDD treatment guidelines recommend considering many factors during initial treatment selection, such as patient symptoms and comorbidities, medication side effect profiles, and patient preference [6]. These guidelines suggest close monitoring of therapeutic benefit and tolerability during the first 4-6 weeks and then throughout treatment, incorporating structured measures of symptom severity, side effects, treatment adherence, and functional status to help assess response to treatment [6,7]. Systematic assessments and a strong therapeutic alliance between provider and patient remain key for tailoring a treatment plan to match the unique needs of each patient, highlighting the importance of measurement-based care (MBC) and shared decision-making (SDM).

### Measurement-Based Care and Shared Decision-Making

MBC includes treatment adjustment evaluation using quantitative measures of symptoms, level of functioning, and quality of life while treating patients with MDD [7]. American Psychiatric Association clinical guidelines recommend MBC during initial screening as well as for monitoring depression throughout treatment [6]. By quantifying clinical outcomes, MBC provides guidance for timely treatment reassessment and modifications tailored to the individual needs of the patient and has been shown to improve outcomes and quality of care [8]. This systematic approach to treatment may have positive effects on the patient-provider relationship and enhance SDM. MDD often requires long-term antidepressant treatment; however, this is challenging for many patients because of barriers such as short consultation times with their care teams and limited involvement in the treatment decision-making process. An increased focus on MDD-relevant patient education, including clarification of the benefits of a specific treatment plan and alignment of such a plan with the patient’s values, can help overcome treatment barriers. Primary care providers can incorporate MBC and play a major role by involving patients with MDD in the SDM process. In addition, SDM can foster patient-provider engagement and increase patient satisfaction [9].

### Digital Tools in MDD

#### Overview

Many mobile apps have recently been developed and are available for the management of depression. Patients with MDD and their treatment providers may benefit from the use of these apps to assist in disease management. However, the majority of these apps have been assessed only in the research setting and have not been integrated into the clinical workflow. Most apps do not undergo a rigorous evaluation of interventions and instead rely on evidence-based theory for design; this may result in limited evidence of efficacy, leading to potential patient misinformation. Attrition is another major challenge for many depression management apps because of poor personalization to improve user-based engagement [10]. In addition, many mobile apps are only patient-facing and have not incorporated a care team interface, and the use of tracked content including symptoms and engagement with interventions in a single app is rare [10]. Creation of user-friendly mobile apps that incorporate user-centered design with input from patients with MDD and health care providers (HCPs) [11] can improve the development process, iteration speed, and implementation success, with the potential to improve patient outcomes in MDD [12-14].

#### Pathway App and Pathway Platform for Patients With MDD

Takeda Pharmaceuticals U.S.A., Inc., Lundbeck LLC, and Advocate Aurora Health (AAH) have partnered to cocreate a digitally enabled care experience with users (patients and care teams) and software developers, as well as health-tech product development specialists (Ctrl Group / Fora Health) to improve patient-provider engagement in the management and treatment of MDD. A digital mobile patient interface—the *Pathway app*—was designed with a conversational texting interface to allow patients to record daily mood symptoms, function, medication adherence, and side effects [11]. The *Pathway app* captures changes in a patient’s response to antidepressant pharmacotherapy and includes standard outcome measures, such as the 9-item Patient Health Questionnaire (PHQ-9) for depression [11]. Patient interaction with the *Pathway app* allows for increased patient awareness of symptoms and overall functioning [11]. Through the *Pathway app*, up-to-date information can be shared with the patient’s HCP to guide clinical discussions and enhance SDM between patients and their clinicians at routine visits as well as between visits, with the aim of optimizing individualized treatment plans and improving overall outcomes [11].

#### Testing of Pathway App in a Pilot Feasibility Study

The *Pathway app* was tested within a real-world pilot feasibility study (ClinicalTrials.gov NCT03242213) in patients with MDD who had recently started or undergone a switch to antidepressant monotherapy [15]. Patients were randomized 1:1 to the control arm (standard of care; n=20) or the app arm (standard of care plus the *Pathway app*; n=20) [15]. The first iteration of the *Pathway app* included patient-reported outcomes (PROs) related to depression, well-being, cognitive symptom tracking, medication adherence, and side effects. The *Pathway app* enabled patients to track their symptoms, monitor their treatment progress, and manually share the data collected with their care team. Results from the pilot study confirmed the effectiveness and feasibility of using the *Pathway app* in patients with MDD and showed a trend toward improved patient engagement in the app arm, albeit in a small sample size [15].

#### Pathway App Follow-up Interviews (Round 1) With Patients and HCPs

After the pilot study, 2 rounds of semistructured qualitative research interviews were conducted by a study team with expertise in qualitative research and user-centered design methods; results were determined using thematic analysis based on a structural coding framework. Round 1 interviews were performed to obtain feedback from patients and HCPs to understand their experience and perceptions of current and new *Pathway app* features to help ensure creation of a more user-centered design [15]. The user-centric approach created an opportunity for continued reassessment based on user feedback, faster iterations, optimized system performance, and sustainability. It also helped enhance the usability of the system, with the ultimate goal of improving treatment outcomes by supporting an expanded understanding of MDD treatment, assisting care team workflows, and providing people living with depression additional support throughout their treatment journey [11]. New features were included in the *Pathway app* that were not part of the pilot feasibility study, such as HCP visit preparation, patient education, and goal setting.

Patients reported that the ability to prepare for HCP visits using the *Pathway app* helped reduce their anxiety, improved the ease of communication with their HCPs, and enhanced the efficiency and effectiveness of appointments. Patients also found that the goal-setting feature of the *Pathway app* helped them focus on their goals and find a constructive purpose. Among the feedback from HCPs on the patient education feature of the *Pathway app,* many commented that it reduced their burden and was visually appealing and engaging.

#### Development of the Pathway Platform

**Figure 1:**
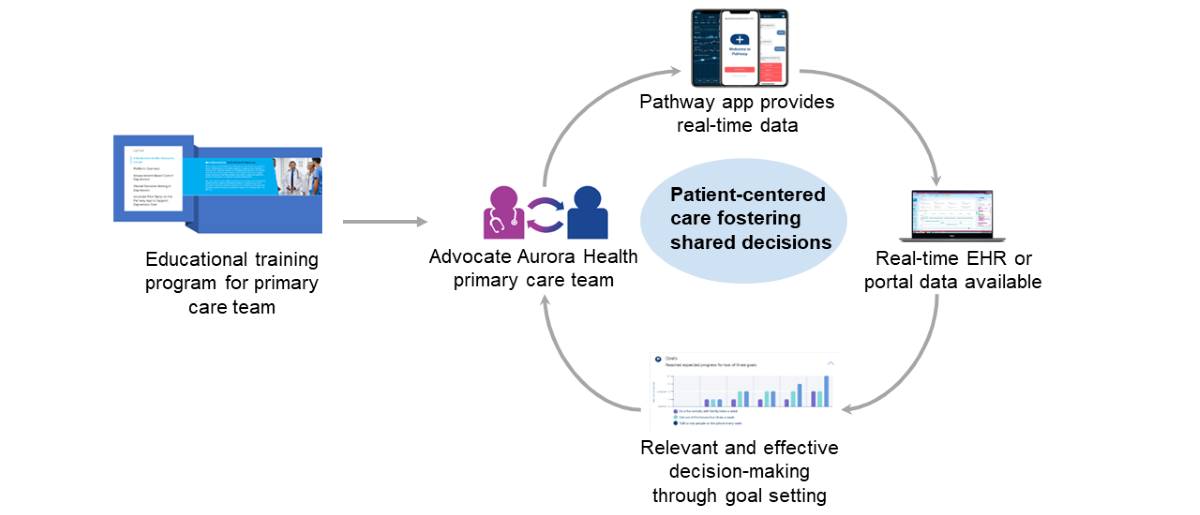
Design of the Pathway Platform. EHR: electronic health record.

Data from the round 1 qualitative interviews gathered from the *Pathway app* pilot feasibility study pointed to the need for enhanced rapid communication of real-time data shared with the care team, integration of the app into the MDD care pathway within the electronic health record (EHR), and education of the care team about usage of these data [11,13]. Building on these results, the *Pathway Platform* was created, which includes a newer iteration of the *Pathway app*, real-time integration of patient data with the Epic EHR (Epic Systems Corporation), as well as an app-based education program for patients and a web-based educational training program for the care team (Figure 1).

#### Pathway Platform Follow-up Interviews (Round 2) With HCPs

Round 2 of qualitative research interviews was conducted with HCPs to understand workflow, usability, and clinical use of the *Pathway Platform*. An ongoing partnership with care teams provided an opportunity for continuous reassessment of the *Pathway Platform* based on user feedback. Key insights included the importance of clinical visit preparation and data visualization for SDM, collaborative goal setting and tracking for patient engagement, and optimization of the educational support tools for both patients and care teams [15]. This feedback was used to add value to the *Pathway Platform* for all users and optimize features prior to the clinical integration study presented here.

### Rationale and Aim of the Study

In this ongoing, large-scale, real-world implementation study, it is our aim to test the scaling and integration of the digitally enabled *Pathway Platform* along with educational interventions at multiple primary care sites within the AAH system [11] and assess its impact on MBC and other evidence-based processes (ClinicalTrials.gov NCT04891224). The primary objective of this study is to determine whether implementing the *Pathway Platform* in the primary care setting improves adherence to MBC through PHQ-9 usage. Secondary objectives are to determine improvement in the additional clinical process measures as well as MDD remission and response outcomes in patients using the *Pathway Platform*.

## Methods

### Study Design

**Figure 2:**
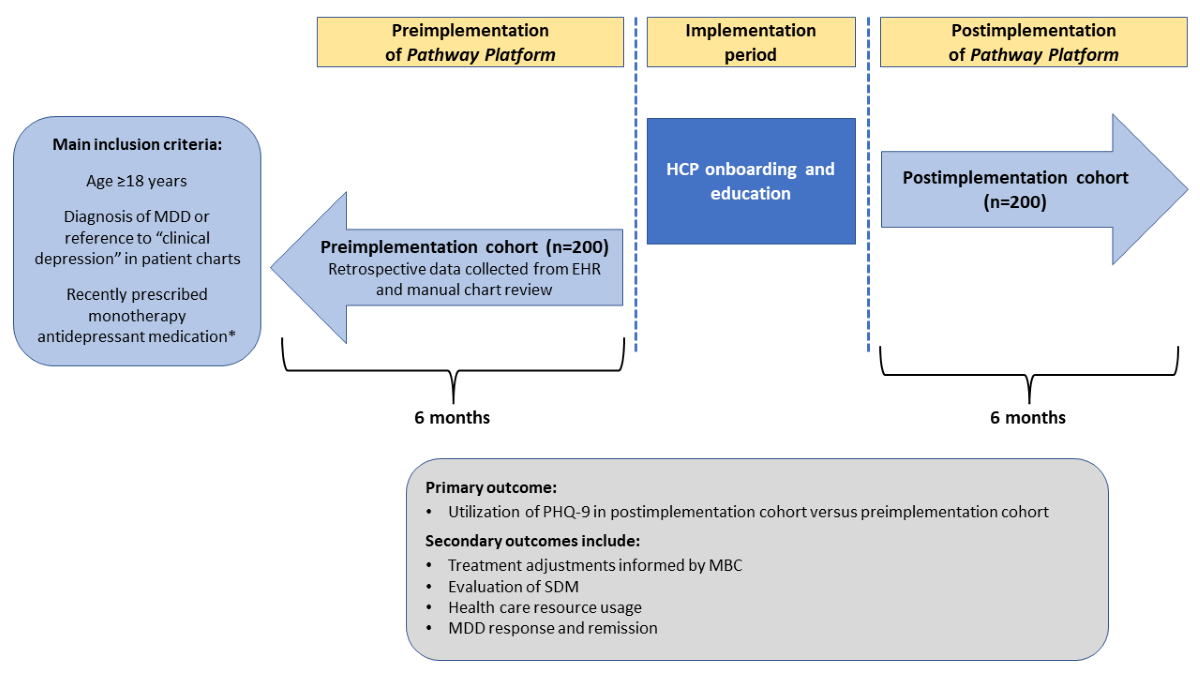
Schematic representation of the real-world implementation study. EHR: electronic health record; HCP: health care provider; MBC: measurement-based care; MDD: major depressive disorder; PHQ-9: 9-item Patient Health Questionnaire; SDM: shared decision-making. *New start, medication switch, or dose change in the past 3 months.

This real-world interventional study uses a pre- and poststudy design to assess the impact of implementing the *Pathway Platform* in the primary care setting (Figure 2) [11]. Regarding implementation of the *Pathway Platform*, the study design is divided into 3 periods: preimplementation, implementation, and postimplementation.

#### Preimplementation

The preimplementation cohort consists of patients who meet the inclusion criteria for enrollment in the study and have visited one of the participating clinics at least 6 months prior to implementation of the *Pathway Platform*. Study outcomes will be assessed using retrospective data collection, including chart reviews and electronic data pulls 6 months prior to implementation of the *Pathway Platform*.

#### Implementation

The implementation period will entail educating care team members before the enrollment visit and onboarding. To confirm eligibility, patient history will be evaluated for 3 months prior to enrollment. During the implementation period, the care team members involved in MDD management will receive education on evidence-based clinical practices for depression care, such as MBC and SDM. Training will be provided for patient onboarding to the *Pathway Platform* and usage of the EHR to view data collected in the platform.

#### Postimplementation

The postimplementation cohort consists of up to 200 patients from the same participating clinics who meet the eligibility criteria and provide written informed consent to download the *Pathway app*. In this cohort, data on patient outcomes will be collected prospectively over 6 months with the option to continue using the app at the 6-month mark until a common study end date (data collected beyond 6 months will be used for exploratory analysis). At the end of 6 months, retrospective EHR and manual chart reviews will be conducted, and performance related to MBC and SDM will be assessed. Clinical performance and selected patient outcomes will be compared before and after implementation of the *Pathway Platform*. Additional patient outcomes will be assessed after implementation among patients with MDD using the *Pathway Platform*.

### Study Sites and Centers

This study is being performed within up to 20 primary care clinics in the AAH care system and will include up to 400 patients with MDD. Advocate is the largest HCP organization in Illinois, with 12 hospitals, 20 health centers, and more than 250 care sites. It is one of the largest accountable care organizations in the United States, caring for more than 2 million patients annually. General practitioners at AAH provide diagnoses, treatment, and clinical care for adults aged ≥18 years experiencing chronic diseases, with MDD often being included among those. Sites representing different geographic locations, patient populations, and care models for depression management, including behavioral health integration models, will be identified to participate in the study.

### Ethical Considerations

This study will be conducted in accordance with legal and regulatory requirements, and the protocol was reviewed by the AAH institutional review board prior to patient recruitment or data collection (approval number AHC-6680-75000249). Because this is designed as a real-world evidence study, investigators and study team members will not be physically located at the sites routinely, so all patients will provide consent through an e-consent process.

### Study Population

All trained care team members who manage patients with MDD within the participating AAH clinics will be invited to participate in this implementation study and will have access to a web-based educational resource center and an app-based educational program for patients.

### Inclusion and Exclusion Criteria

Eligible patients aged ≥18 years, diagnosed with MDD based on *International Classification of Diseases, Tenth Revision*, codes or who have a reference to “clinical depression” in their patient charts, and have recently (past 3 months) been prescribed monotherapy antidepressant medication or had a recent switch (past 3 months) in medication or medication dose, will be included in this study. Patients should be able to use the *Pathway Platform* based on clinician’s judgment (eg, own an Apple iPhone version 5 or later version or a smartphone with an Android operating system and have an active data plan or regular Wi-Fi access).

To be eligible, patients should have tolerability concerns or an inadequate response determined by a PHQ-2 (includes the first 2 questions of the PHQ-9, which cover the degree to which a patient has experienced depressed mood and anhedonia within the past 2 weeks) score ≥3, or a PHQ-2 score <3 with a PHQ-9 score of ≥5 recorded in medical records in the past 6 weeks or during screening [16]. Patients with a missing PHQ-2 score in the past 6 weeks or who are unable to perform PHQ-2 during the enrollment visit; who have been diagnosed with bipolar depression, schizophrenia, or schizoaffective disorder; or who are no longer under primary care for depression (have transitioned to a psychiatric care team) will be excluded from this study.

### Study Procedures and Measurements

The *Pathway Platform* consists of 3 components: the latest iteration of the *Pathway app*, EHR integration, and educational scaffolding.

#### Pathway App

**Figure 3:**
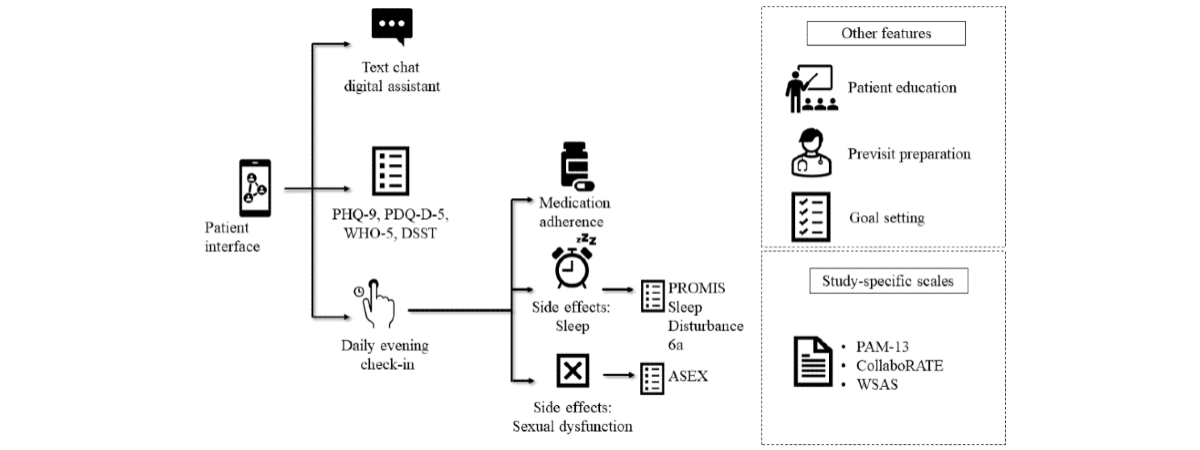
Features of the *Pathway app*. ASEX: Arizona Sexual Experience Scale; DSST: Digit Symbol Substitution Test; PAM-13: 13-item Patient Activation Measure; PDQ-D-5: 5-item Perceived Deficits Questionnaire–Depression; PHQ-9: 9-item Patient Health Questionnaire; PROMIS: Patient-Reported Outcomes Measurement Information System; WHO-5: 5-item World Health Organization Well-being Index; WSAS: Work and Social Adjustment Scale.

This study will deploy the most recent iteration of the *Pathway app*, which prompts patients to complete the following 4 scales every 2 weeks: PHQ-9 and 5-item Perceived Deficits Questionnaire–Depression (PDQ-D-5) to assess depression status, 5-item World Health Organization Well-being Index (WHO-5) to assess quality of life, and Cognition Kit Digit Symbol Substitution Test to assess cognition [17-20]. The *Pathway app* also includes a daily evening check-in to collect information on medication adherence and side effects; patients reporting side effects related to sleep will be prompted to complete the Patient-Reported Outcomes Measurement Information System (PROMIS) Sleep Disturbance item 6a, and patients reporting side effects related to sexual dysfunction will be prompted to complete the Arizona Sexual Experience Scale (ASEX). There will be an optional function to set and track goals using the goal attainment scale adapted for depression during clinical visits with an HCP [21]. Patients are also provided with a feature they can use to prepare for an upcoming visit with their care team, along with other features (Figure 3).

For purposes of evaluating this implementation study, 3 additional scales assessing patient-provider engagement and functional improvement will be deployed at baseline and at the end of the study (13-item Patient Activation Measure [PAM-13], CollaboRATE [also included as a 3-month postenrollment assessment], and Work and Social Adjustment Scale [WSAS]) [22-24]. During the study, if a patient’s response to item 9 of the PHQ-9 indicates a change in their suicidal ideation, they will receive a pop-up notification to contact their HCP or emergency services immediately. A similar instruction is also given in routine clinical practice. Patients will also be informed during the consent process that the information provided to the *Pathway Platform* is not directly or continuously monitored in real time by care teams.

#### EHR Integration

Data collected by the *Pathway app* will be electronically transmitted and stored in the patient’s EHR. The data will be accessible to the care team and provide a longitudinal summary that may assist in SDM and depression management. Providers can view the data either before or during the patient visit and use it to discuss future depression management.

#### Educational Scaffolding

A web-based educational training program for primary care team members was developed using evidence-based medicine, building on the concepts of MBC and SDM as they relate to depression management. This education resource center houses a series of videos where key leaders in depression treatment and management from psychiatry and primary care speak about the strategies to optimize patient outcomes and suggest practical applications of the *Pathway Platform* in primary care along with supporting evidence from the pilot study. Care team members are asked to review the videos before the first patient is enrolled, which will be tracked to evaluate the proportion of providers who completed the education. Additionally, a guide for the care team is available. The training program includes a care provider audit and feedback where provider performance related to PHQ-9 use will be presented during the study period. An app-based education program along with a training manual was developed for patients, which describes the functionality of the *Pathway Platform* and how to use and interpret the data collected.

### Study Outcomes

Evaluation of the success of the *Pathway Platform* implementation will be performed by comparing study outcomes among both cohorts (pre- and postimplementation). The primary outcome will be usage of PHQ-9 in the postimplementation cohort versus the preimplementation cohort over a 6-month period. The primary end point will be the change in the proportion of patients with at least 2 PHQ-9 scores before and after implementation. Secondary outcomes will include treatment adjustments informed by MBC, evaluation of SDM, MDD remission and response, and health care resource usage, including referrals to behavioral health and primary care follow-up after hospitalizations. Results from PRO scales, such as PAM-13, CollaboRATE, and WSAS will be assessed to compare baseline and 6-month follow-up data among patients using the *Pathway Platform*.

Exploratory outcomes, including medication adherence, goal attainment, and PHQ-9, PDQ-D5, and WHO-5 scores, will be evaluated among patients who use the *Pathway Platform*. Safety assessments will include adverse events related to the *Pathway Platform*. No adverse events are expected in this minimal-risk study; however, all serious adverse events that are spontaneously reported during the study period will be collected. Causality with study procedures will be determined for all serious adverse events and deemed “related” or “not related” to the intervention.

### Statistical Analysis Plan

The following hypotheses will be examined: (1) PHQ-9 use will be greater after implementation compared with before implementation of the *Pathway Platform* and (2) provider processes and patient depression-related outcomes will have greater improvements after implementation of the *Pathway Platform* compared with previous practice patterns. For primary and secondary hypothesis testing, process measures, patient clinical outcomes, and health care resource usage will be compared before and after implementation of the *Pathway Platform*. Additional PROs will be evaluated after implementation only, which will be from patients using the *Pathway Platform* at baseline and at the end of the study.

For preimplementation, outcomes will be assessed among patients who visited one of the study clinics at least once over the preimplementation study period and were eligible to use the *Pathway Platform* if it had been available. For the postimplementation period, the *Pathway Platform* will be the data source for paired *t* test analysis, assessing change in continuous variables, including PHQ-9 scores. Categorical outcomes will be presented as proportions and compared using the Pearson chi-square test. Continuous outcomes will be assessed for normality and presented as means and SD or medians and IQRs; continuous outcomes will be compared using the Student *t* test for independent groups. Two-tailed tests using a significance threshold of *P*=.05 will be used for all tests.

### Data Collection and Management

The schedule of evaluations is given in Table 1. Data from the EHR and manual chart reviews will be collected from two cohorts: (1) patients attending clinics after implementation (postimplementation cohort) and (2) patients attending clinics before implementation (preimplementation cohort). For each cohort, data will be collected over a 6-month period. Data on patient outcomes and *Pathway Platform* usage will be collected throughout the study starting on day 1 through month 6 for each patient using the platform. Similarly, data on report usage by the care team will be collected throughout the study. In addition, the number of times the care team educational resource material is viewed will be recorded between day 1 and month 6.

**Table 1:** Schedule of postimplementation evaluations.

Study day	Baseline assessment (day 1)		Final assessment (month 6)	
	EHR^a^	*Pathway Platform*	*Pathway Platform*	EHR
Demographic characteristics	✓	—^b^	—	—
Number of PHQ-9^c^ scores documented	✓	—	✓	✓
Treatment adjustments	✓	—	✓	✓
Referral to behavioral health	✓	—	—	✓
PHQ-9 scores	✓	—	✓	—
ED^d^ visits	✓	—	—	✓
ED visits due to mental illness	✓	—	—	✓
Hospital admissions	✓	—	—	✓
Follow-up after ED and hospital visits	✓	—	—	✓
Hospital admissions due to mental illness	✓	—	—	✓
Outpatient visits, including phone calls	✓	—	—	✓
Outpatient visits, including phone calls due to mental illness	✓	—	—	✓
Remission and response (using PHQ-9 data)	✓	—	✓	—
PAM-13^e^	—	✓	✓	—
CollaboRATE	—	✓	✓	—
WSAS^f^	—	✓	✓	—
PHQ-9	—	✓	✓	—
WHO-5^g^	—	✓	✓	—
PDQ-D-5^h^	—	✓	✓	—
DSST^i^	—	✓	✓	—
Medication adherence	—	✓	✓	—
Side effects	—	✓	✓	—
PSD-6^j^ (only if reporting side effects of sleep problems)	—	✓	✓	—
ASEX^k^ (only if reporting side effects of sexual problems)	—	✓	✓	—
Goal setting	—	✓	—	—
Goal attainment	—	—	✓	—
App analytics	—	✓	✓	—
Report analytics	—	✓	✓	—
Educational material analytics	—	✓	✓	—
AEs^l^ or SAEs^m,n^	✓	✓	✓	✓

^a^EHR: electronic health record.

^b^—: Not available.

^c^PHQ-9: 9-item Patient Health Questionnaire.

^d^ED: emergency department.

^e^PAM-13: 13-item Patient Activation Measure.

^f^WSAS: Work and Social Adjustment Scale.

^g^WHO-5: 5-item World Health Organization Well-being Index.

^h^PDQ-D-5: 5-item Perceived Deficits Questionnaire–Depression.

^i^DSST: Digit Symbol Substitution Test.

^j^PSD-6: Patient-Reported Outcomes Measurement Information System (PROMIS) Sleep Disturbance 6a.

^k^ASEX: Arizona Sexual Experience Scale.

^l^AE: adverse event.

^m^SAE: serious adverse event.

^n^During the conduct of the study, adverse events or serious adverse events will be reported. As such, reports will be spontaneously notified, and causality of any AEs will be assumed unless there is evidence to the contrary.

## Results

Patient recruitment was completed in July 2022, with initial results expected in mid-2023. Findings are expected to provide insights into the improvements that can be made in clinical workflows to enhance collaborative care, depression management, clinician and patient experience, adherence to medication, patient-provider engagement, and depression outcomes in the primary care setting for patients with MDD [11]. EHR integration and efficiencies with current AAH information technology platforms will also be assessed [11].

## Discussion

Primary care centers are significant providers of mental health services for patients with MDD. Among US adults with MDD, 33% reported receiving mental health treatment within primary care during the previous 12 months compared with 21% who received treatment from a psychiatrist [25]. MBC in patients with MDD allows the primary care provider to accurately identify whether a patient is making enough progress toward their treatment goals. Treatment decisions based on MBC give patients a better understanding of the disease, including how adherence impacts their symptoms [8].

A common barrier to adherence is when an HCP’s choice of treatment plan is not agreed upon by the patient. SDM is a systematic approach that improves patient education and helps primary care physicians provide treatment in conformity with evidence-based guidelines [26,27]. SDM can help increase adherence to drug treatment in the primary care setting and results in improved social functioning compared with usual care when used as part of a multifaceted relapse prevention program [9,28]. Involvement in SDM is associated with a higher probability of receiving quality care and improvement in symptoms [29].

Digital technology platforms, such as mobile apps, can offer low-cost interventions to monitor and improve services for certain patient populations that are difficult to retain in treatment and may also promote better health [30]. The *Pathway Platform* is a digital platform designed to improve patient outcomes by supporting an expanded understanding of MDD treatment, assisting care team workflows, and providing additional support to patients with depression throughout their treatment journey [11,15]. The patient-facing interface includes a digital assistant that allows those with MDD to interact with the *Pathway Platform* in a chat-based conversational text interface. The information collected from a patient using the *Pathway Platform* is intended to assist the care team in managing the patient’s depression through improving MBC and SDM, thereby enhancing patient-provider engagement and ultimately patient outcomes.

The uniqueness and strengths of this study design are the real-world implementation of a digitally enabled care pathway with EHR integration in real time for patients with MDD and a continuous educational program for both patients and care teams in the primary care setting. In addition, the care team interface and patient-provider engagement help incorporate the use of PROs and emphasize the importance of SDM. Integrating the *Pathway Platform* to support MBC and SDM will improve the clinical workflow, depression management, adherence to medication, patient-provider engagement, as well as depression outcomes and care.

SDM and the involvement of both patients and physicians promote healthy relationships, with the benefit of helping patients with MDD feel greater support throughout their treatment, including web-based connections and care when needed, and can help ensure the safety of everyone involved in the process. By cocreating the *Pathway Platform* with users, including user-centered design methods, extensive preimplementation feedback and optimization, incorporation of the care team workflow, and patient preferences, the chances for successful implementation are greater. From a digital perspective, the *Pathway Platform* has the potential for eventual scalability and sustainability outside a research setting. Clear documentation of the study design and implementation methods will allow for replication at other health care institutions.

Limitations include a smaller sample size and geographically limited study centers, which prevent broader generalizability. Additionally, the use of a pre- and postevaluation design and lack of randomization can be seen as sources of accidental bias.

Data obtained from this real-world implementation study will allow for functional assessment of the *Pathway Platform* within the AAH care system and will determine its effects at a primary care level in patients with MDD. The *Pathway Platform* has the potential to improve patient-provider engagement for patients diagnosed with MDD and will address whether engaging these patients on an ongoing basis using digital tools can lead to better health outcomes. Overall, the study is planned to demonstrate the value of an appropriate MDD clinical care pathway by providing useful insights into a high-impact health care system.

## Abbreviations

AAH: Advocate Aurora Health
ASEX: Arizona Sexual Experience Scale
EHR: electronic health record
HCP: health care provider
MBC: measurement-based care
MDD: major depressive disorder
PAM-13: 13-item Patient Activation Measure
PDQ-D-5: 5-item Perceived Deficits Questionnaire–Depression
PHQ-9: 9-item Patient Health Questionnaire
PRO: patient-reported outcome
PROMIS: Patient-Reported Outcomes Measurement Information System
SDM: shared decision-making
WHO-5: 5-item World Health Organization Well-being Index
WSAS: Work and Social Adjustment Scale

## Data Availability

This study is ongoing. Further information regarding the study design is available from the corresponding author upon reasonable request.
